# Weibo sentiments and stock return: A time-frequency view

**DOI:** 10.1371/journal.pone.0180723

**Published:** 2017-07-03

**Authors:** Yingying Xu, Zhixin Liu, Jichang Zhao, Chiwei Su

**Affiliations:** 1 School of Economics and Management, Beihang University, Beijing, China; 2 Department of Finance, Ocean University of China, Qingdao, Shandong, China; East China University of Science and Technology, CHINA

## Abstract

This study provides new insights into the relationships between social media sentiments and the stock market in China. Based on machine learning, we classify microblogs posted on Sina Weibo, a Twitter’s variant in China into five detailed sentiments of anger, disgust, fear, joy, and sadness. Using wavelet analysis, we find close positive linkages between sentiments and the stock return, which have both frequency and time-varying features. Five detailed sentiments are positively related to the stock return for certain periods, particularly since October 2014 at medium to high frequencies of less than ten trading days, when the stock return is undergoing significant fluctuations. Sadness appears to have a closer relationship with the stock return than the other four sentiments. As to the lead-lag relationships, the stock return causes Weibo sentiments rather than reverse for most of the periods with significant linkages. Compared with polarity sentiments (negative *vs*. positive), detailed sentiments provide more information regarding relationships between Weibo sentiments and the stock market. The stock market exerts positive effects on bullishness and agreement of microblogs. Meanwhile, agreement leads the stock return in-phase at the frequency of approximately 40 trading days, indicating that less disagreement improves certainty about the stock market.

## Introduction

Early research on stock market prices, e.g., random walk theory and Efficient Market Hypothesis (EMH) suggest that stock prices are driven by new information that is unpredictable [1, 2]. Later studies, however, present the contrary evidence, supporting that stock market prices can indeed to a certain degree be predicted [3] and casting doubts on the EMH. Furthermore, emotions and mood of financial agents affect investment decisions [4, 5], thus resulting in changes in stock markets. A growing body of research suggests that early indicators can be extracted from online social media (e.g., blogs, Twitter feeds) to predict changes in the stock market [6]. In contrast, [7] identifies that sentiments regarding stock markets have no discernible effect on stock prices.

Research that examines whether and how microblogging messages are related to stock indicators appears to present promising results in the United States, Eastern Europe, and Germany [8, 9]. For example, [10] finds that certain mood states on Twitter (www.twitter.com) are related to the closing values of the Dow Jones Industrial Average (DJIA). The study [6] reports a high correlation between stock prices and Twitter sentiments and Granger causalities running from Tweet sentiments to stock prices. [11] explores data from StockTwits, a microblogging platform exclusively dedicated to the stock market, and documents that the posting volume can improve the forecast of trading volume in the stock market. Experimental results in [12] demonstrate that sentiments of Tweets with cash-tags, which indicate that corresponding Tweets are related to stocks, help improve the prediction of stock returns markedly. Although certain evidence is provided [13, 14, 15], to the best of our knowledge, the relationship between microblogging messages and stock markets remains empirically understudied in China. In contrast to most emerged markets, the marketing policy intervention in China will introduce non-market factors that may disturb the stock market. Moreover, those possible interventions can be leaked through social media and then affect investors’ sentiments and decisions. Given that there is a significant quantity of individual investors in China, online news is likely to impact the stock market as indicated by the conventional behavioral finance theory. Indeed, [16] demonstrates that the establishment of Sina Weibo, one of the most popular social networking platform, as an alternative information interaction channel has changed the overall stock market behavior. The evolution of the Internet has made it easier to obtain interactive data and has created new channels for information interaction. Therefore, the relationship between sentiments extracted from Sina Weibo and the stock market performance may provide certain evidence regarding the prediction of stock prices [17]. Moreover, with the wild evolutions of social media and stock markets, relationships between sentiments of microblogs and stock markets are not likely to maintain unchanged in recent years. This paper makes the following contributions to the literature.

First, in contrast to related previous studies that assume a constant relationship between sentiments of online social media and the stock market, this study considers possible changes in both time and frequency domains in the relationship. Previous literature, in general, refers to methods of the Pearson correlation and Granger causality tests that test unchangeable relationships between series. Thus, those results are susceptible to misleading conclusions in the presence of changes in the relationships. Investigations based on different time horizons may come to contrary conclusions. This paper adopts a Wavelet Coherency Analysis based on the continuous wavelet transformation to revisit the relationship between sentiments extracted from Sina Weibo (www.weibo.com) and the stock market.

Second, data based on Chinese microblogs from Sina Weibo, a Twitter-like service, help weaken the sample-bias. Most previous studies focus on microblog postings from Twitter that are in English. As Twitter’s user base becomes increasingly international, the bias induced by geographical and cultural sampling errors is unavoidable [10]. Sina Weibo launched in China in 2009 and accumulated over 500 million users in fewer than four years. Most users locate in China and post Chinese microblogs, thus weakening the bias of sampling errors.

Finally, most previous studies categorize microblogs into positive and negative sentiments, ignoring more detailed dimensions. The oversimplification of the emotion classification makes it difficult to disclose detailed correlations of specific sentiments. Negative emotions such as anger, disgust or sadness are more applicable in real world scenarios, i.e., abnormal event detection or emergency tracking [18]. Thus, figuring out the relationships between detailed sentiments and stock markets may shed light on understanding how abnormal events affect financial markets through the network.

This paper collects 157,351 microblogs regarding Chinese stock markets from June 2014 to December 2014, and classifies the sentiment of an individual microblog into five categories, including anger, disgust, fear, joy, and sadness. To compare with previous research, we also categorize emotions into negative and positive and construct two indices of Bullishness and Agreement. We focus on relationships between various sentiments and the stock market and examine how such relationships change over time and across different scales (frequencies). To do so, we utilize the continuous wavelet analysis which can localize correlations between series and evolution in time and across scales. Both time and frequency are important for stock price movements considering that the Chinese stock market has undergone a wild evolution in recent years. It is reasonable to believe that relationships between sentiments of microblogs and the stock market have changed. Additionally, the frequency domain viewpoint provides an opportunity to distinguish between short- and long-term correlations. We show that relationships between sentiments of microblogs and the Chinese stock market are not always significant, particularly at high frequencies. The most significant relationship appears surrounding ten trading days, and sadness has a closer relationship with the stock return than the other four sentiments. Compared with polarity sentiments (positive and negative), detailed sentiments show more persistent effects. Bullishness has a loose and unstable relationship with the stock market. Regarding the lead-lag relationship, the agreement of investors leads the stock market in-phase over the long run. However, we find that the stock market leads detailed and polarity sentiments rather than the reverse. Our results suggest that relationships between Weibo sentiments and the stock market change over time and across frequencies and detailed sentiments contain more information regarding the stock market than polarity sentiments. Furthermore, causalities running from agreement to the stock return indicate contrary evidence regarding the EMH.

## Literature review

Over the past decade, significant progress has been made in sentiment tracking techniques that extract indicators of public mood directly from social media content, such as Internet stock message boards like Yahoo! Finance, in particular large-scale Twitter feeds and microblogs [18, 19]. [20] is the first to investigate the relationship between internet stock message boards and stock market activities. Using a sample of over 3,000 stocks listed on Yahoo! message boards, the study finds that changes in daily posting volume are associated with earnings-announcement events and daily changes in stock trading volume and returns. Consequently, the volume of messages predicts changes in next day stock trading volume and returns. [21] examines the relationship between the Internet message board activity and abnormal stock returns and trading volume based on the RagingBull.com discussion forum. They report that changes in investor opinions correlate with abnormal industry-adjusted returns and abnormally high trading volume on days with eccentrically high message activity. No such linkage is found between the message board activity and industry-adjusted returns or the trading volume, which is consistent with the EMH. [22] categorizes stock recommendations (buy, sell, and neutral) through a database under Google’s ownership, and suggests that these recommendations are overwhelmingly positive. Nevertheless, no evidence supporting that the recommendations have new information is found, indicating no significant linkage between internet stock messages and stock market activities. [23] establishes that stock microblog sentiments do have predictive power for simple and market adjusted returns using microblog postings from StockTwits and Yahoo! Finance, respectively.

The above studies focus on readily available quantitative information such as the message volume and user ratings and do not capture the information content and sentiments of microblog messages. As emphasized in [19], newsworthiness may be linked to tones, positive or negative. They identify a gatekeeping function, implying that newsworthiness may be associated with negativity across a wide range of subjects. The finding is buttressed by the literature on individuals’ disproportionate attentiveness to negative versus positive information, across many subjects, e.g., [24, 25]. These literature suggest that negative information is in certain situations viewed as being more important than equally positive information. Similarly, [26] finds that negative words contain more information than positive words.

Thus, contents and sentiments implied in messages from social media are captured by automated classifiers and are used to predict stock market activities. Most studies appeal to the binary sentiment (positive vs. negative) analysis. For instance, by employing an online sentiment classifier called Twitter Sentiment Tool (TST), [27] detects sentiments of Tweets as positive and negative. Based on the Granger causality analysis, they use sentiments and the consumer confidence in the product to address the problem of predicting daily up and down movements in individual tech stock prices. Their results indicate that causalities running from Tweet sentiments to stock prices exist. The study [13] assigns the respective label (positive or negative) for each Tweet and demonstrates that it holds significant predictive power for subsequent stock market movements. [28] uses a supervised machine learning approach to train a sentiment classifier, and categorizes a Tweet into one of the given classes (positive or negative sentiment). By using the Granger causality test, they show that positive and negative sentiments indicate stock price movements a few days in advance. Using a regression analysis, [29] demonstrates a significant relationship between a specific firm’s stock return and the cumulative emotional valence (positive or negative) of Tweets regarding the firm. [30] finds a relatively low Pearson correlation and a Granger causality between the sentiment regarding the 30 stock companies (positive or negative) posted on Twitter and the corresponding Dow Jones Industrial Average (DJIA) index over the entire period. However, the result based on “event-study” shows that the sentiment polarity during the peaks of Twitter volume implies the direction of cumulative abnormal returns. Thus, polarity sentiments appear to contain useful information regarding stock activities.

Sentiment analysis tools used in the literature adhere to a binary distinction between positive and negative sentiments. Nevertheless, rich and multi-dimensional structures of human moods were ignored [10]. The Google-Profile of Mood States (GPOMS), a mood analysis tool employed in [10] captures human mood states regarding six different mood dimensions, namely alert, calm, happy, kind, sure, and vital. They establish that the mood time series identified by GPOMS respond to significant socio-cultural events such as the Presidential Election and Thanksgiving. According to the results of Granger causality tests and Self-Organizing Fuzzy Neural Network, they conclude that the calm sentiment identified by GPOMS is predictive of the DJIA. By contrast, general levels of positive and negative sentiments measured by another mood analysis tool of Opinion Finder cannot predict the DJIA. In other words, if there is a great deal of “calm” emotion in Tweets on a given day, the DJIA tends to rise over the following two to five days.

Whereas these studies have investigated the relationships between online social media sentiments and stock markets, most are based on data from emerged markets such as the United States and Europe. With the establishment of Sina Weibo, the microblogging web-site dominator in China [31], more focus is put on the Chinese market. [32] shows positive relationships between investors’ bullish sentiment indices from Sina Weibo and stock market indices using Granger causality tests and impulse responses. [33] uses a sentiment analysis technology to automatically classify reviews from Sina Finance, a typical financial website related to Sina Weibo, as positive or negative, and then identifies investor sentiment as either bullish or bearish. Their empirical results suggest solid correlations between stock price volatility trends and stock forum sentiments. [34] shows that the bullish and bearish stock market conditions extracted from Sina Weibo articles have strong correlations with the Shanghai Composite Index (SHCI) particularly when the data set has distinguishing characteristics. [31] documents that stocks with high original and re-post microblogs are often accompanied by low average volumes per transaction, implying a close linkage between microblogs and the stock market. Using Harvard psychological dictionary and Loughran—McDonald financial sentiment dictionary, [35] constructs a sentiment space and suggests that sentiments from news articles improve the prediction of Hong Kong Stock Exchange prices. Nevertheless, simply focusing on positive and negative dimensions cannot bring useful predictions. [36] obtains five sentiments including anger, disgust, fear, happiness, and sadness by analyzing the text content of daily microblogs from Sina Weibo using an emotion lexicon. Results of the Granger causality analysis and linear regression suggest that the sadness sentiment improves the predictive accuracy of trading volume. In the study of [37], the results based on correlation analysis and causality test show that the stock market in China can be competently predicted by Weibo emotions, e.g., disgust, fear, joy, and sadness.

The linkage between sentiments of online social media and financial markets is assumed to be constant in the above investigations, which is not likely to be true. The following studies provide evidence that contradicts the constant relationship. [38] reveals that 71% of the messages posted on Twitter do not travel any farther than the authors’ timeline, thus indicating that most of the information is hardly propagated through the network. Based on machine learning techniques and the inference of time-dependent diffusion probabilities from a multidimensional analysis of individual behaviors, [39] illustrates two peaks in the transmissions of Tweets. There is a quick outbreak of the information at the beginning, and the transmission appears to die out before another peak of activity occurs. Furthermore, [40] finds that approximately 73% of topics have a single active period and 15% and 5% of topics have two and three active periods, respectively. Very few have more than three active periods. Consequently, sentiments generated by posts on social media are more likely to have structural changes and the linkage between sentiments and stock markets is time-varying. [32] provides certain direct evidence, in which the results of impulse response show that short-term effects of investors' bullish sentiment indices of posts on Sina Weibo on stock returns are significant. In a related study, [41] applies a continuous wavelets framework and finds that the Bitcoin price is positively related to search queries on Google and searched words on Wikipedia. Furthermore, the relationship changes over time and frequency. Therefore, we explicitly investigate the time-varying and frequency-related linkage between sentiments of stock related microblogs on Sina Weibo and stock market activities in China using the wavelets analysis.

## Methodology

### Sentiment classification

We accumulate 157,351 microblogs with keywords relating to Chinese stock markets from June 2014 to December 2014 by crawling posts on Sina Weibo through its open Application Programming Interfaces (APIs) under the authority granted by Sina Weibo. The content of microblogs is recorded in the form of short text which is limited to 140 characters. Many approaches have been proposed to mine sentiments from texts in recent years. For example, the lexicon-based method is widely used. [42] uses human evaluations of the emotional content of individual words within a given text to quantify the overall score of happiness levels on large-scale texts. By employing Amazon Mechanical Turk, [43] scores over 10,000 English words on an integer scale from one to nine, where one represents sadness, and nine represents happiness. Using data from millions of public Twitter messages, [44] identifies individual-level mood rhythms using Linguistic Inquiry and Word Count (LIWC). Another widely used method is machine learning based solution. [45] runs classifiers trained on emoticon data against a test set of Tweets (which may or may not have emoticons in them) which are classified as either positive or negative. In contrast to most works which categorize emotions into negative and positive, [18] employs emoticons for the generation of sentiment labels for microblogs on Sina Weibo and builds an incremental learning Naive Bayes classifier for the categorization of four types of sentiments: angry, disgust, joy, and sadness.

This paper adopts the method in [38, 46] to classify sentiments of microblogs from Sina Weibo. Numerous training samples are required to handle the extremely short text in microblogs. Considering that both smiley and emoticons are strongly related with typical sentiment words and are viewed as convincing indicators of emotions [47], we treat these emoticons as sentiment labels. The most commonly used 95 emoticons are selected for the labeling stage. Several experts label their emotions manually based on the images of emoticons and approximately 50 frequent words occurring together with emoticons. We find that most of the emoticons are labeled by five sentiments, including anger, disgust, fear, joy, and sadness. For other emotions such as surprise, insufficient votes are found. Thus, we classify the sentiments of microblogs as five classes.

Approximately 10,000 microblogs with valid emoticons are extracted and labeled. Using the labeled emoticons as a training corpus *C*_*tr*_, we build a fast Bayesian classifier to mine the sentiment of microblogs without emoticons. For each microblog *t* in *C*_*tr*_, we convert it into a sequence of words {*W*_*i*_}, where *W*_*i*_ is a word, and *i* is its position in *t*. The prior probability of the world *W*_*i*_ belonging to the sentiment category *m* is provided by:

$$\text{P}\left(W_{i}/m\right)=\frac{n^{m}\left(W_{i}\right)+1}{\sum_{q}\left[n^{m}\left(W_{q}\right)+1\right]}$$ (1)

where *m = 1*, *2*, *3*, *4*, *5*, *n*^*m*^*(W*_*i*_*)* denotes the times that *W*_*i*_ appears in all microblogs in the category *m*, and the Laplace smoothing is used to avoid the problem of zero probability. For an unlabeled microblog *t* with word sequence {*W*_*i*_}, its category can be obtained as

$$m^{*}\left(t\right)=\text{arg }max_{m}P\left(m\right)\Pi_{i}P\left(W_{i}/m\right)$$ (2)

where *P(m)* is the prior probability of *m*. We set *P(m) = 0*.*2* to guarantee the overall recall of the classifier. Using Eq (2), we can classify microblogs without emoticons into five sentiments. [18] trains a fast Naive Bayes classifier on data from Sina Weibo for emotion classification, and the system named MoodLens whose vital part is the emotion classifier is now available online for temporal and spatial sentiment patter discovery.

### Wavelet analysis

The wavelet transform is a commonly used signal conversion and processing method, which can be traced back to the Fourier analysis. Wavelets are localized in both time and frequency domains and allow for analysis of time-frequency dependencies between two time series. In the case of the wavelet transform, the time resolution is intrinsically adjusted to a frequency with the window width narrowing when focusing on high frequencies whereas widening when assessing low frequencies. Furthermore, wavelets are useful for processing non-stationary time series and extracting information of time domain localization [48]. Compared with commonly used approaches of the Pearson Correlation and the Granger Causality test [12, 13], the wavelet analysis can identify the time-varying relationships between microblog sentiments and stock returns, and it considers the frequency domain [41].

Wavelet Transformation is a process of decomposition and superposition of information. By adding a series of basis wavelet which is obtained by translation and dilation of the so-called mother wavelet, the original time series is transformed into a two-dimensional plane of time-frequency. The continuous wavelet transform (CWT) *W*_*x*_*(τ*, *s)* is obtained by projecting the mother wavelet *ψ(t)* into examined time series *X(T)*. The mother wavelet is defined as:

$$\psi_{\tau ,s}\left(t\right)=\frac{1}{\sqrt{s}}\psi \left(\frac{t-\tau }{s}\right)$$ (3)

where *τ* denotes the location parameter which measures the time position of the mother wavelet, and *s* is the scale parameter which indicates how the wavelet is stretched or dilated. If *s<*1, the wavelet is compressed and captures rapid changes at high frequencies. If *s>*1, the wavelet is stretched and captures slow changes at low frequencies. Given a mother wavelet, the CWT is defined as:

$$W_{x}\left(\tau ,s\right)=\int_{-\infty }^{+\infty }x\left(t\right)\psi_{\tau ,s}{}^{*}\left(t\right)\;dt$$ (4)

where *ψ*_*τ*, s_*(*t*) represents the complex conjugate of the basis wavelet *ψ*_*τ*, *s*_*(t)*. Consequently, the wavelet transform decomposes a time series *x(t)* regarding certain basis wavelets that are obtained by translation and dilation of the mother wavelet *ψ(t)*. The most commonly used mother wavelet in analyzing information on both amplitude and phase is the Morlet wavelet [49], which is a complex sine wave within a Gaussian envelope:

$$\psi_{\omega_{0}}\left(t\right)=\pi^{-1/4}\left(e^{i\omega_{0}t}-e^{-\omega_{0}{}^{2}/2}\right)\;e^{-t^{2}/2}$$ (5)

In Eq (5), *π*^-1/4^ ensures that the wavelet function has unit energy, and $e^{-\omega_{0}{}^{2}/2}$ ensures the admissibility condition of a mother wavelet. If *ω*_0_>5, $e^{-\omega_{0}{}^{2}/2}$ can be ignored [50]. In this case, the Morlet wavelet is simplified as follows:

$$\psi \left(t\right)=\pi^{-1/4}e^{i\omega_{0}t}\;e^{-t^{2}/2}$$ (6)

The parameter *ω*_0_ in Eqs (5) and (6) denotes the wavenumber, which measures the number of oscillations within the Gaussian envelope. There are two conflicting objectives in choosing *ω*_0:_ frequency localization and time localization. A greater *ω*_0_ is accompanied by a better frequency localization but a poorer time localization, and vice versa. As noted in [40], *ω*_0_ is usually set to six to guarantee a good balance between time and frequency resolution.

The wavelet power spectrum of a time series *x(t)* is defined as the modulus square of the CWT, i.e. │*W*_*x*_*(τ*, *s)*│^2^, which recovers the relative contribution at each time and each scale to the time series variance. The wavelet power spectrum can be integrated across *τ* and *s* to recover the total variance in the series:

$$\sigma_{x}{}^{2}=\frac{1}{C_{\psi }}\int_{0}^{+\infty }\int_{-\infty }^{+\infty }{\left|W_{x}\left(\tau ,s\right)\right|}^{2}\frac{d\tau ds}{s^{2}},\text{ with }0<C_{\psi }=\int_{0}^{+\infty }\frac{{\left|\hat{\psi }\left(\omega \right)\right|}^{2}}{\omega }\text{d}\omega <\infty$$ (7)

where $\hat{\psi }\left(\omega \right)$ is the Fourier transform of *ψ(t)*. The cross-wavelet transform of two time series *x(t)* and *y(t)* is defined as *W*_*xy*_*(τ*, *s) = W*_*x*_*(τ*, *s)W*_y_*(*τ*, *s)* [51]. The cross-wavelet spectrum is correspondingly defined as │*W*_*xy*_*(τ*, *s)*│^2^ = │*W*_*x*_*(τ*, *s)*│^2^│*W*_*y*_*(τ*, *s)*│^2^, implying the local co-movement between *x(t)* and *y(t)*.

The wavelet coherency coefficient measures the local strength of the relationship between two series over time and across frequencies.

$${\text{R}}^{2}\left(\tau ,s\right)=\frac{{\left|S(s^{-1}W_{x,y}\left(\tau ,s\right))\right|}^{2}}{S(s^{-1}{\left|W_{x}\left(\tau ,s\right)\right|}^{2})S(s^{-1}{\left|W_{y}\left(\tau ,s\right)\right|}^{2})},\text{ with }{\text{R}}^{2}\left(\tau ,s\right)\in \left[0,1\right]$$ (8)

where *S(∙)* is the smooth factor in time and scale normalization. Without smoothing, the coherency is identically one at all scales and times. Smoothing is achieved by convolution in time and scale. More details please refer to [48]. *s*^-1^ converts to an energy density [52]. The value of *R*^*2*^*(τ*, *s)* should be within the interval [0, 1]. The greater value of wavelet coherency coefficient indicates the stronger comovement between *x(t)* and *y(t)*. Based on a large number of Monte Carlo simulations, [53] documents that the wavelet power spectrum asymptotically follows the *χ*^2^ distribution with two degrees of freedom. As a consequence, we can test the significance of the correlation by testing the null and alternative hypothesis as follows:

$$H_{0}:\;\left[\frac{\left|W_{x}\left(\tau ,s\right)W_{y}{}^{*}\left(\tau ,s\right)\right|}{\sigma_{x}\sigma_{y}}\right]\leq \frac{3.999}{2}\sqrt{P_{f}^{x}P_{f}^{y}}$$ (9)

$$H_{1}:\;\left[\frac{\left|W_{x}\left(\tau ,s\right)W_{y}{}^{*}\left(\tau ,s\right)\right|}{\sigma_{x}\sigma_{y}}\right]>\frac{3.999}{2}\sqrt{P_{f}^{x}P_{f}^{y}}$$ (10)

where *P*_*f*_^*x*^ and *P*_*f*_^*y*^ are the background spectra of *x(t)* and *y(t)* under the *χ*^2^ distribution. If the null hypothesis *H*_*0*_ is rejected, we interpret the result as *x(t)* and *y(t)* are correlated with each other under the significance of 5%. If the null hypothesis cannot be rejected, we tend to believe that the correltion between the two indices are insignificant.

To investigate the lead-lag relationships, we use the wavelet phase difference between *x(t)* and *y(t)* [53]. The wavelet phase difference is defined as the ratio of the imaginary component of the cross-wavelet transform *W*_*xy*_*(τ*, *s)* to the real part:

$$\phi \left(\tau ,s\right)=arc\;tan\left(\frac{I\left(s^{-1}W_{xy}\left(\tau ,s\right)\right)}{R\left(s^{-1}W_{xy}\left(\tau ,s\right)\right)}\right),\text{ with }\phi \left(\tau ,s\right)\in \left[-\pi ,\pi \right]$$ (11)

Graphically, the phase difference is represented by an arrow. If the arrow points to the right (left), the series are in the in-phase (anti-phase), meaning that they are positively (negatively) correlated. If the arrow points down (up), *x(t)* leads *y(t)* by *π/2* (vice versa). Usually, the relationship is a combination of the two. For example, if the arrow points to the northeast, the series are positively correlated, and *y(t)* leads *x(t)*. Note that the interpretation of phase relationships is partially dependent on specific expectations regarding the relationship because a leading relationship in the in-phase can be a lagging relationship in the anti-phase.

## Data

We have collected 157,351 stock-related microblogs of 12 to 3,292 daily postings; this represents an average of 1,299 posts per trading day with a standard deviation of 623 messages. Using the classification method explained previously, we classify microblogs into five categories, i.e., anger, disgust, fear, joy, and sadness. Furthermore, as a comparison, we count the numbers of posts with the sentiment of joy as positive microblogs and the remaining as negative microblogs for each day of the time series. As suggested in [54], it is the natural starting place to test whether the level of microblogs or the bullishness of them affect stock markets. The second issue is whether a greater disagreement is associated with the stock market. The financial theory provides two distinct perspectives on disagreement. Whereas [55] and others hold the hypothesis that disagreement induces trading, the “no-trade theorem” [56] argues that disagreement leads to a revision of stock prices. Following the literature [6, 11, 57], we define the proxy of Bullishness for each day as:

$$Bullishness_{t}=\text{ln}\left(\frac{1+M_{t}^{Positive}}{1+M_{t}^{Negative}}\right)$$ (12)

where *M*_*t*_^*Positive*^ and *M*_*t*_^*Negative*^ denote the number of positive or negative microblogs on a particular day *t*. The index of Bullishness measures the share of surplus positive signals and gives more weight to a greater number of microblogs in a specific sentiment (positive or negative). The agreement among positive and negative microblogs is represented by:

$$Agreement_{t}=1-\sqrt{1-{\left(\frac{M_{t}^{Positive}-M_{t}^{Negative}}{M_{t}^{Positive}+M_{t}^{Negative}}\right)}^{2}}$$ (13)

The value of *Agreement*_*t*_ is greater if more agents share the same sentiment. If all microblogs regarding the stock market are of the same sentiment, Agreement would be one in that case. The Shanghai Composite Index (SHCI) denotes the average stock price of Shanghai Stock Market. Thus, we use the stock return based on the closing price of SHCI as the index of the stock market. The data are available in Wind database.

We test the correlation between microblog sentiments and the stock return in Table 1. According to the Pearson Test, which tests the linear relationship between two variables, the numbers of microblogs with detailed sentiments exhibit different linkages with the stock market. Fear has a positive linear relationship with the return of SHCI, indicating that more fear sentiments are likely to increase overall stock returns of the Chinese market. However, no linear relationship is found in other sentiments. The non-linear relationship tests of Kendall’s τ_b test and Spearman correlation test suggest that fear and sadness sentiments and Bullishness are positively related to the return of SHCI. The agreement of posts on Sina Weibo shows a negative linkage with the stock market in three tests. The positive relationship between Bullishness and the stock market indicates that more microblogs sharing the positive sentiment are accompanied by a greater SHCI return. The result agrees with the behavioral economics hypothesis suggesting that when people are happy and optimistic, they are more likely to increase investment, which in turn improves the stock market performance [13]. On the other hand, the results of fear and sadness sentiments imply a contrary conclusion, suggesting that more such pessimistic sentiments are accompanied by better stock performance. Furthermore, the correlation between Agreement and SHCI return is significantly negative, indicating that if agents are more concentrated in one sentiment, the stock market is likely to have a lower return. The results of linear and nonlinear correlation tests support close linkages between microblog sentiments and the stock market for the full sample period, in line with previous literature [58, 59, 60]. However, the results provide no information regarding the lead-lag relationships between social media sentiments and the stock market.

**Table 1:** Correlations between microblog sentiments and the stock return.

	Stock Return		
	Pearson test	Kendall's τ_b test	Spearman test
***Anger***	0.112	0.025	0.033
***Disgust***	0.087	0.086	0.116
***Fear***	0.198**	0.106*	0.150*
***Joy (Positive)***	0.068	0.000	0.006
***Sadness***	0.114	0.109*	0.163**
***Negative***	0.093	0.055	0.075
***Agreement***	-0.202**	-0.112**	-0.173**
***Bullishness***	0.127	0.106*	0.163**

Notes:

** and * denote significance at the 5% and 10% level, respectively. These tests are used by Stata software. The results of positive sentiment are the same as joy.

**Fig 1:**
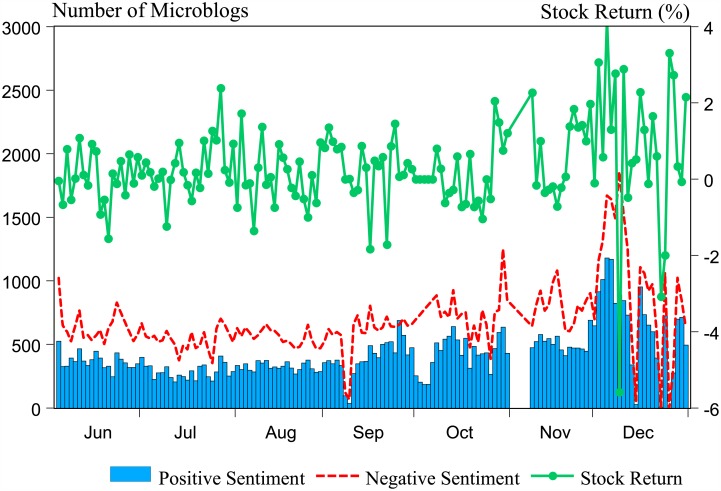
Positive and negative microblogs and the SHCI return.

**Fig 2:**
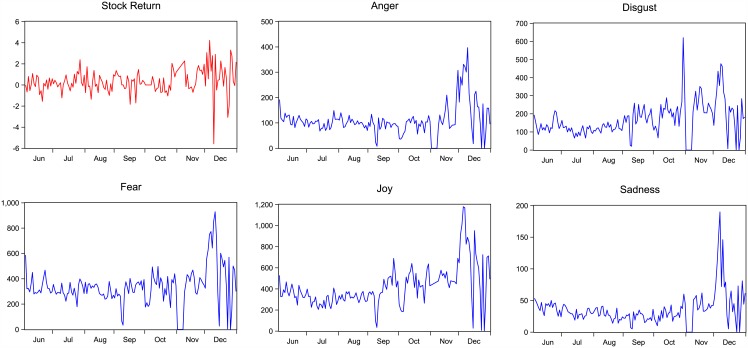
Microblogs with anger, disgust, fear, joy, sadness sentiments and the SHCI return.

The proposed visual presentation of sentiment time series and stock return can be seen in Figs 1 and 2. Peaks show the days when people intensively posted microblogs regarding the stock market. Two prominent negative peaks can be observed in October and December, and one positive peak shows up in December. This type of visualization can be used as a tool for easier and faster overview analysis of general observation of trends. It is interesting to observe that the correlations between microblog sentiments and the stock return are not fixed. For example, the sentiments are comparatively stable in the first half sample and are mostly negative. As sentiment reversed its polarity in September, remarkable fluctuations appeared in the second half of the sample. Analogously, the return of SHCI fluctuated more widely during October to December. If we focus on the contemporary movement between sentiments and the stock return, positive linkages are likely to be observed. For instance, during September 29th to October 17th, the stock return shares the same trends with both positive and negative sentiments. However, in the following month, the stock return moves in the opposite direction of negative sentiment. Meanwhile, positive sentiment coordinates the movement of stock return. Thus, the relationship between sentiments and the stock market is not constant and appears to be different across frequencies.

More specific changes in sentiments can be observed from Fig 2. A significant decrease in anger can be found surrounding November 21st, when the central bank of China announced a cut in the interest rate for the first time in the past 28 months, thus inducing a steep increase in the capital market. At the end of October, when half stocks encountered sharp decreases, and the other half increased significantly, microblogs with disgust and joy sentiments reached comparatively high levels and accounted for approximately 70% of all posts. When the daily trading volume exceeded 1.07 trillion Yuan on December 5th, most investors were excited and posted approximately 1,169 microblogs with joy sentiment. By contrast, the abnormal performance of the stock market triggered certain investors’ concern and fear about the future market. Many agents were worried about the short-term correction of the stock market and its departure from the real economy. Consequently, the sentiment of fear appeared a high level. Subsequently, this concern appears to come true with the stock return decreased to negative on December 9th and disgust and fear reached peaks. On the evening of December 9th, the Securities Registration of China announced the measures about strengthening risk management of bond repurchase, which was viewed as a black swan of the bond market. Consequently, the SHCI receded approximately 9% from the peak among 49 months and decreased 5.4% in one day. Meanwhile, the negative sentiment posted on Sina Weibo reached a peak. During December 3rd to December 15th, investors were suffered from the fluctuating stock market and showed peaks and troughs in all sentiments. The changes suggest that sentiments extracted from Sina Weibo are closely related to the return of SHCI and captures significant breaks of the Chinese stock market.

## Empirical results

### Morlet wavelet power spectra

**Fig 3:**
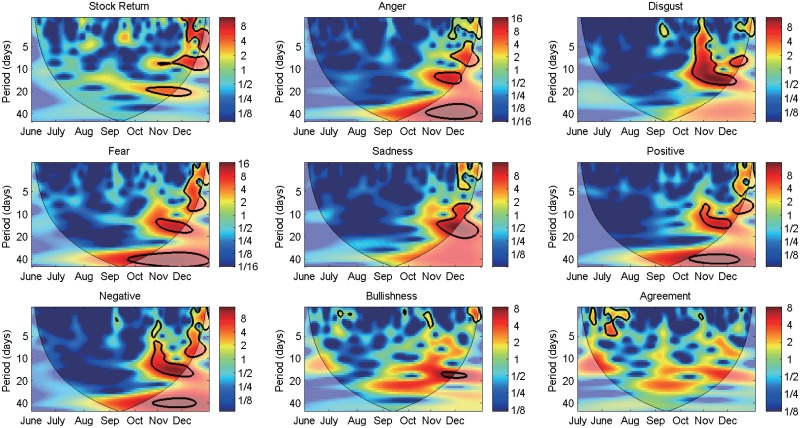
Morlet wavelet power spectra of sentiments and the SHCI return.

Fig 3 presents the Morlet wavelet power spectra of the return of SHCI, microblog sentiments, Bullishness, and Agreement. The horizontal axis denotes time, and the vertical axis represents different scales (or frequency) (i.e. the wavelet transform periods). We categorize time scales of fewer than five trading days as short-term, those between 5–20 trading days as medium-term, and those more than 20 trading days as long-term. The black contour areas represent significance at the 95% confidence interval. High power value of a certain variable is represented by highlighted red area, suggesting that the spectral energy is high, and vice versa. The high power areas indicate the impulse from events, showing that the variable fluctuates significantly. The cones formed with bold black lines bending upward denote the Cones of Influence (COI), representing the boundary conditions of the Morlet wavelet transform. The CWT assumes that the data are circulatory, thus making certain bias at the beginning and end of the sample period in addressing finite-length time series. Thereby, outer regions of the COI indicate that fluctuations in variables are sensitive to the edge effect.

The wavelet spectra provide a first assessment of the individual behavior of data in a time and frequency varying framework. The wavelet power spectrum of stock return shows no significant fluctuation for most of the sample period before November. During November to December, the return of SHCI records three regions of high power spectra at frequencies of fewer than five and 5–20 trading days, suggesting that the spectra are different among distinct time-frequency spaces. As with the stock return, five sentiments show significant fluctuations among both time and frequency domains. Most high power spectrum regions appear surrounding November, and detailed sentiments fluctuate significantly over medium to high frequencies, matching the intense short to mid run cyclical oscillations in the time series during that period. No significant fluctuation is found at low frequencies except for fear. Negative sentiment shares a similar pattern with anger and disgust and appears to merge idiosyncratic characteristics of more detailed sentiments. The movements of Bullishness and Agreement are moderately different from other sentiments with the fluctuations in both indices being less persistent and less pervasive. For instance, Agreement fluctuates significantly at frequencies of fewer than five trading days in August. Bullishness is comparatively stable over the entire sample period. Similar fluctuations in five sentiments and the stock market are captured in November and December, but distinct characteristics exist.

### Coherence analysis

**Fig 4:**
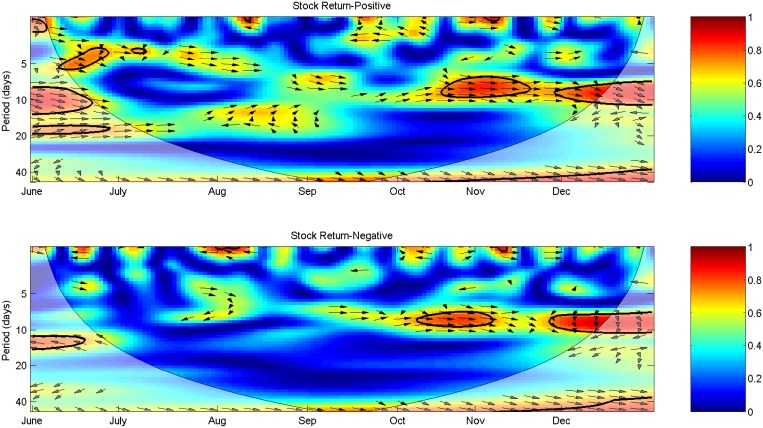
Morlet wavelet transform cross-spectra and coherence spectra of polarity sentiments and the SHCI return.

The coherence and phase relationship of microblog sentiments and the stock return illustrate the co-movement and lead-lag relationships between indices. Fig 4 shows the Morlet wavelet transform cross-spectra and coherence spectra of polarity sentiments and the SHCI return. In Fig 4, the color code for coherency ranges from blue corresponding to low coherency (close to zero) to red corresponding to high coherency (close to one). The thick black contour denotes the 5% significance level estimated from a Monte Carlo simulation of the wavelet coherency between 10,000 sets of two white noise time series with the same length studied in this paper. Within the designated areas the time series are significantly correlated at the 5% significance level. Analogously, there is a COI in cross-spectral power, representing the boundary conditions, and results within COI should be interpreted with caution. The arrows imply the phase difference between series as defined in Eq (11). Through Fig 4, we can identify both frequency bands (in the vertical axis) and time intervals (in the horizontal axis) where two indices move together. Moreover, we can explain the extent of the correlation between them across time and frequencies.

We note several findings from analyzing the co-movement patterns presented in Fig 4. First, for positive and negative sentiments, significant relationships occur primarily at medium scales and since mid-October. At higher frequencies, the significant regions are short-lived and can be due to statistical fluctuations and noise [41]. For the lead-lag relationship, it is the stock return that leads positive and negative sentiments in-phase. Fig 4 shows a significant relationship between positive sentiment and the stock market during October 20th to November 4th at the frequency of 5–10 trading days. At the beginning of October, when other peripheral markets, e.g., the United States, Europe, and Japan were undergoing frequent changes, Chinese stock markets were closed because of the National Day holiday (October 1st to 7th). Therefore, negative sentiments were pervasive since the holiday and investors were worried about the trend of Chinese markets. During this period, fear sentiment accounted for 30% of total posts on Sina Weibo. Nevertheless, out of the widespread falling expectation, the SHCI maintained a relatively stable level, thus leading to the superficial positive effect of the stock return on negative sentiment. Hence, during this phase, the actual causation of negative sentiment is more likely to be the downtrends of peripheral markets rather than the performance of the Chinese stock market. The positive linkage between negative sentiment and the return of SHCI disappeared when China Securities Regulatory Commission announced the schedule of the Shanghai-Hong Kong Stock Connect. This landmark program provides a cross-boundary investment channel between Shanghai and Hong Kong stock markets. On October 26th, Shanghai-Hong Kong Stock Connect was postponed officially, which was interpreted as bull news by many microblogs because it implies a speculative opportunity. On the following day, the market of United States routed, followed by an increase rather than a decrease of the SCHI. The excessive rise stimulated investors’ positive sentiments, and the prediction that Chinese stock market is stepping into a new adjustment which signals a bull market was pervasively re-posted on Sina Weibo.

The effect of the stock market on positive sentiment sustained to November 17th, when Shanghai-Hong Kong Stock Connect was launched officially and failed to stimulate the SHCI in approximately three trading days. According to the posts on Sina Weibo, although the temporary decline of the SHCI has dampened positive sentiments inevitably, many investors insisted that Shanghai-Hong Kong Stock Connect heralds a secular bull market. With the return of SHCI increased on November 20th, positive sentiments rose significantly. The leading role of the stock market in affecting positive sentiment is strengthened by the uptrend period of late November to December, during which the daily return of SHCI reached 1.8%. However, the surge raised fear sentiments significantly with many posts expected a sharp fall to be followed. Consequently, movements in the return of SHCI since late November exert effects on both positive and negative sentiments. Whereas the positive sentiment exerts a significant instant effect on the SHCI return in June at the frequency of fewer than five trading days, no such linkage is found with negative sentiment. The result contradicts the conclusion that negative microblogs are more influential than positive microblogs in China [46] and negative posts contain more information and are more related to the stock market [26], at least from the view of stock relevant microblogs. One possible reason may be the fact that Chinese investors appear to be more overconfident than those in the United States, making them believe that past returns are indicative of future returns and increasing the trading volume and stock returns [61].

**Fig 5:**
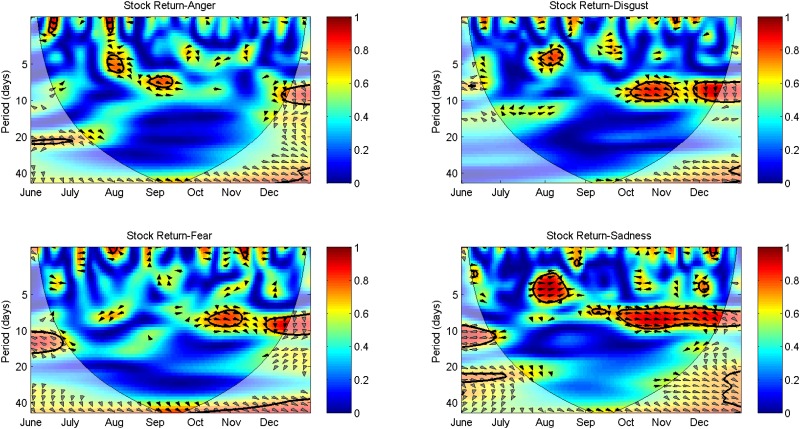
Morlet wavelet transform cross-spectra and coherence spectra of detailed sentiments and the SHCI return.

About the relationships between detailed sentiments and the stock market, we find considerable variation in relationship patterns across sentiments. Sadness shows a strong relationship with the stock market at high to medium frequencies throughout the sample period. As shown in Fig 5, significant co-movements between sadness sentiment and the return of SHCI are found during July 23th to August 20th at the frequency surrounding five trading days, and since September 5th at the frequency surrounding ten trading days. As indicated by the directions of arrows, the short-run fluctuations in sadness sentiments extracted from microblogs lead the movement of the stock market in phase during July 23th to August 20th. The medium-run fluctuations in the stock market are synchronously related to sadness with a comparatively small positive phase difference since September 5th. This result suggests that the return of SHCI is a leading indicator of sadness sentiment over the second half of the sample period. This effect may be due to the dynamic fluctuations in peripheral markets as analyzed previously. Anger presents a different pattern with less persistent co-movement with the stock market. During two short periods, i.e., July 28th to August 11th and August 28th to September 11th, the return of SHCI leads anger in phase. According to the mental accounting theory, faced with the sharp rise and fall in stock returns, investors are easy to segregate small gains from large losses and integrate small losses with large gains, leading to significant increase in sadness sentiments [46, 62]. Consequently, it appears that sadness exerts a closer relationship with the stock market than anger. The result conflicts with [46], in which anger sentiment is found to be more influential than sadness. Nevertheless, it is consistent with the conclusion of [36], in which sadness extracted from Sina Weibo performs better in predicting the SHCI volume than other sentiments of anger, disgust, fear, and happiness. Similarly, [37] finds that anger extracted from Sina Weibo has the weakest correlation with the Chinese stock market.

The case of fear, where the stock market acts as a leading indicator is observed mainly at the medium frequency of ten trading days, providing a similar pattern as negative sentiment, particularly during October 20th to November 14th and since November 26th. As with fear, significant relationships between disgust and the stock market appear during October 13th to November 7th and since November 26th. Affected by the fluctuating peripheral markets during Chinese National Day holiday, investors were pessimistic about the trend of SHCI. In the following 20 trading days, the peripheral markets kept fluctuating and strengthened the linkages between pessimistic sentiments and the stock market. Analogously, Shanghai-Hong Kong Stock Connect intensified the leading role of the SHCI return in affecting fear and disgust sentiments. The results are in contrast to [8] which argues that calm is the sole sentiment that causes the value of DJIA and other sentiments (alert, sure, vital, kind, and happy) have no effect on stock prices. Our analysis provides evidence that sadness has a certain leading role in affecting the stock market, and different sentiments have distinct relationships with the return of SHCI that are time-varying and frequency related.

**Fig 6:**
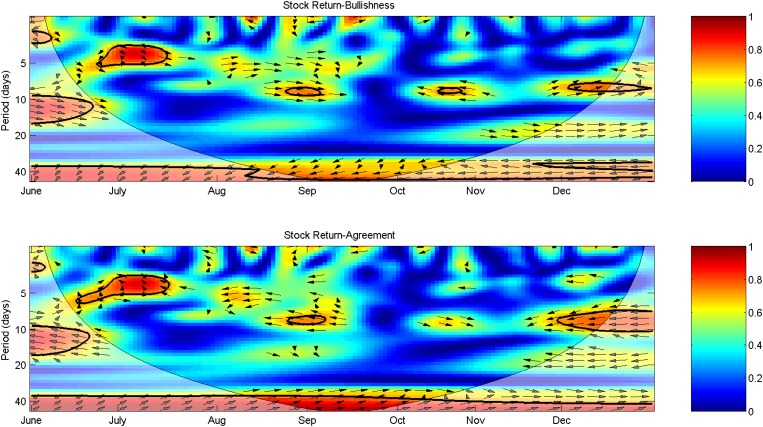
Morlet wavelet transform cross-spectra and coherence spectra of Bullishness/Agreement and the SHCI return.

Individual investors are considered the least informed market participants [63]. For informed agents with limited investment capacity, they are motivated to spread informative but imprecise stock tips so that followers trade on the advice and move prices as they wanted [64]. In particular, investors are often influenced by word of mouth [65, 66, 67], reflecting their bounded rationalities in trading. Consequently, they subject to a persuasion bias, thus generating a positive relationship between the stock return and Bullishness indicator which measures the share of surplus positive signals and gives more weight to a greater number of positive posts [68]. Along with the theoretical properties, Fig 6 shows a positive phase difference between Bullishness and the SHCI return since December at the frequency surrounding ten trading days. However, no such linkage is observed over other periods. During June 25th to July 16th and August 28th to September 4th, the SHCI return leads Bullishness indicator in phase at the frequency of fewer than five trading days and ten trading days, respectively. Moreover, Bullishness leads the stock market anti-phase surrounding October 28th. Thereby, the relationship between Bullishness and the stock market is loose and unstable.

As suggested in [69], disagreement about market information leads to extensive debate and release of more information. Substantial disagreement implies that agents are uncertain about stock markets, thus causing the trading volume to rise because participants assign different values to assets [55]. Conversely, more information may not only reflect pre-existing information but also have changed market behaviors [70]. Thus, the effect of higher disagreement on the stock market is difficult to predict. Fig 6 shows the linkage between Agreement and the return of SHCI. The negative co-movements at high to medium frequencies are in line with the results of correlation tests in Table 1. During June 23th to July 17th when Agreement indicator fluctuates at the frequency of fewer than five trading days, the linkage between Agreement and the stock market is significant at the same frequency. The arrows point to the northwest, indicating that Agreement indicator leads the SHCI return anti-phase. During this period, more posts convey negative sentiments, agreeing with the indication that more agreement indicates a worse performance of the stock market. Similar results are demonstrated surrounding September. Since December, when the stock market becomes more turbulent, the linkage with Agreement changes and the SHCI return leads the indicator anti-phase. The result suggests that when the stock market is more volatile, increases in stock returns will rise the disagreement in investors. The result is in line with opinions conveyed by posts on Sina Weibo in December. The overgrowth of the SHCI at the beginning of December induced contrary expectations on future trends of the Chinese stock market. Over the long run, Agreement leads the return of SHCI in-phase at the frequency of approximately 40 trading days with arrows pointing to the northeast. The result indicates that agreements on long-run investments rise the performance of the stock market, supporting the view that less disagreement improves market certainty. A similar positive relationship between Agreement and the stock market is found by [57].

Overall, from the perspective of Time Domain, there are significant relationships between sentiments and the stock market in China. Nonetheless, the co-movements are unstable, and patterns of relationships are not consistent across time scales. Specifically, in most of the time during October to December when other peripheral markets are fluctuating, the SHCI return is positively correlated with Weibo sentiments, as suggested by the evidence found in [6] and [71]. Over other periods, such linkage is less persistent. Whereas sadness and Agreement exert a certain leading role in affecting the stock return, the other sentiments have no such effects. From the perspective of Frequency Domain, significant relationships over low to high frequencies are demonstrated, meaning that both short-run and long-run fluctuations in sentiments and the stock market are closely related. Comparatively, the relationships of sentiments and the stock market are stronger at medium frequencies surrounding ten trading days, as indicated by the darker areas shown in the figures. Except for Agreement, other sentiments and indicators show no significant linkages at frequencies of more than 20 trading days. Moreover, results based on Wavelet analysis are in contrast to correlation tests, implying that full-sample results may be misleading and revealing that the relationships between sentiments extracted from Sina Weibo and the stock market are time-varying and frequency related.

## Conclusions

This article classifies microblogs posted on Sina Weibo into five detailed sentiments (anger, disgust, fear, joy, and sadness), two polarity sentiments (positive *vs*. negative), and two indices representing bullishness and disagreement of stock markets (Bullishness and Agreement). Then, we assess the relationships between Weibo sentiments and the stock market in China through wavelet analysis, which is effective in capturing both frequency and time-varying features within a unified framework. There are contradictory results in the literature regarding linkages between sentiments extracted from social media and stock markets. Considering that market participants in emerging markets are more likely to be affected by psychology and sentimentality than those in developed markets, potential relationships between sentiments of investors and stock markets may be found in China. A time-frequency view of relationships between Weibo sentiments and Chinese stock market over seven months suggests that the linkage is not always significant, particularly at high frequencies. Our findings indicate that relationships between microblog sentiments and stock returns have both frequency and time-varying features. On the one hand, the linkage between sentiments and the stock return appears to be stronger at medium-term horizons surrounding ten trading days. Compared with the other four sentiments, sadness has a closer relationship with the stock market. Polarity sentiments have certain significant relationships with the stock market but are less persistent than that of detailed sentiments. On the other hand, the agreement of investors is a leading indicator of the stock market over the long run. However, regarding the lead-lag relationship, it is the stock market that leads detailed and polarity sentiments rather than the reverse. The relationship between bullishness and the stock market is not that significant. According to the results, Chinese investors’ sentiments are likely to be affected by domestic and peripheral market trends and abnormal events. Meanwhile, social media sentiments exert certain effects on Chinese market trends.
